# Overview of Commercial Vaccines Against Canine Visceral Leishmaniasis: Current Landscape and Future Directions

**DOI:** 10.3390/pathogens14100970

**Published:** 2025-09-25

**Authors:** Josiane Aparecida Martiniano de Pádua, Diego Ribeiro, Victor Freire Ferreira de Aguilar, Tuane Ferreira Melo, Lilian Lacerda Bueno, Ana Laura Grossi de Oliveira, Ricardo Toshio Fujiwara, Kelly Moura Keller

**Affiliations:** 1 Departament of Preventive Veterinary Medicine, Veterinary School, Pampulha Campus, Universidade Federal de Minas Gerais (UFMG), Av. Antônio Carlos 6627, P.O. Box 567, Belo Horizonte 31270-901, Brazil; jmartinianovet@gmail.com; 2 Department of Veterinary Clinical, Faculty of Veterinary Medicine and Animal Science, Universidade do Estado de São Paulo (UNESP), St. Prof. Doutor Walter Maurício Correa, P.O. Box 560, Botucatu 18618-681, Brazil; diego.ribeiro@unesp.br; 3 Department of Parasitology, Institute of Biological Sciences, Pampulha Campus, Universidade Federal de Minas Gerais (UFMG), Av. Antônio Carlos 6627, P.O. Box 486, Belo Horizonte 31270-901, Brazil; victorffaguilar10@gmail.com (V.F.F.d.A.); lilacerdabueno@gmail.com (L.L.B.); fujiwara@icb.ufmg.br (R.T.F.); 4 Department of Veterinary Medicine, Faculty of Animal Science and Veterinary Medicine, Universidade Federal de Lavras (UFLA), P.O. Box 3037, Lavras 37200-000, Brazil; tuaneferreiramelo@gmail.com; 5 Faculty of Medicine, Postgraduate Program in Health Sciences: Tropical Medicine and Infectology, Pampulha Campus, Universidade Federal de Minas Gerais (UFMG), Av. Antônio Carlos 6627, P.O. Box 486, Belo Horizonte 31270-901, Brazil; analauragrossi@gmail.com

**Keywords:** public health measures, prevention, zoonotic disease

## Abstract

Visceral leishmaniasis is a zoonosis commonly caused in Brazil by the parasite *Leishmania infantum*. This protozoan parasite can infect several species of mammals, with dogs being the main reservoir in urban areas. Several methods are used to prevent the disease, including collars impregnated with 4% deltamethrin to prevent contact between the sandfly and the animal, and vaccines. Vaccines aim to stimulate an immune response that can effectively fight the parasite, with the Th1 immune response being the most desired. There are several research groups around the world dedicated to testing new immunogens against *Leishmania* spp. and there are currently two commercially available vaccines used to prevent the disease, Neoleish^®^ and Leti-Fend^®^. Leish-Tec^®^, a vaccine previously licensed for use in dogs in Brazil, was suspended in May 2023 due to non-compliance in some batches. This also happened with CaniLeish^®^, which was discontinued by the European Commission in October 2023. These vaccines have different characteristics that influence their use as a public health measure, and therefore the objective of this review is to describe these immunogens, their characteristics, and their use as a collective prevention measure for canine visceral leishmaniasis.

## 1. Introduction

Canine visceral leishmaniasis (CVL) is caused in Brazil by the parasite *L. infantum*, which is transmitted by insects of the subfamily Phlebotominae, mainly the species *Lutzomyia longipalpis*, popularly known as the “straw mosquito,” “birigui,” and other popular names [1]. The protozoan commonly infects cells of the mononuclear phagocyte system [2], and animals that develop clinical signs may present with various skin lesions, onychogryphosis, lymphadenopathy, anorexia, weight loss and cachexia, conjunctivitis, kidney lesions, locomotion problems, among others [3].

Since 2016, the Ministry of Health of the Federal Government of Brazil has permitted the treatment of dogs with the active ingredient miltefosine because the medication is not used in the treatment of human cases of leishmaniasis in the country. This treatment aims to improve the quality of life of dogs by reducing clinical signs and enhancing the efficiency of the immune system to control the parasite, resulting in a reduction in parasite load [4]. However, monotherapy with miltefosine is not recommended, as it carries a high risk of treatment failure or relapse upon completion [5].

Despite the reduction in clinical signs and parasite load in some animals, the therapy does not provide a parasitological cure [6]. For this reason, efforts should focus on prevention measures such as collars impregnated with 4% deltamethrin, which prevent contact between dogs and sand flies, thus averting infection [7]. Collaring is highly effective in reducing the number of canine and human cases of leishmaniasis and, for this reason, it is recommended by the Brazilian Ministry of Health in Technical Note No. 5/2021-CGZV/DEIDT/SVS/MS.

In addition to the use of collars, vaccines are also recommended as prophylaxis against the disease. They aid the immune system in controlling the parasite. For this purpose, vaccines must stimulate an increase in the initial production of interleukins such as IL-12 by antigen-presenting cells and a decrease in the production of IL-10 [8]. This leads to the induction of a strong inflammatory response, with the participation of natural killer cells, CD4+ Th1, and CD8+ lymphocytes. These cells are responsible for increasing concentrations of IL-2, IFN-γ, and TNF-α, thus characterizing the Th1 immune response profile, which is associated with the resistance of animals to the parasite [8]. Therefore, the aim of this review is to describe immunogens, their characteristics, and their use as a preventive measure against CVL.

## 2. Leish-Tec^®^

Leish-Tec^®^ is a Brazilian vaccine composed of the recombinant A2 protein from *Leishmania donovani* amastigotes, together with a saponin adjuvant [9]. The A2 antigen was the first virulence factor identified in *Leishmania* and is essential for the parasite’s survival in mammals [10].

The A2 genes form a widely described family in the *donovani* complex and are expressed in both the amastigote and promastigote phases of the parasite [11]. However, the protein is predominantly abundant in the amastigote form, not in the promastigote, and is primarily located in the cytoplasm [11].

The protein ranges from 42 to 100 kDa and consists almost entirely of 40 to 90 copies of the amino acid sequence Val-Gly-Pro-Leu-Ser-Val-Gly-Pro-Gln-Ser (VGPLSVGPQS), preceded by N-terminal leader sequences [12]. It is highly conserved among *L. infantum*, *L. amazonensis*, and *L. mexicana*, which facilitates the induction of cross-protection between different species [13]. In addition, it is involved in parasite survival under various stress conditions, with production varying within the amastigote population according to the stress levels encountered by parasites within macrophages [14], as reported by McCall and Matlashewski [14]. A2 production is induced, for example, four hours after a heat-shock challenge, suggesting a crucial role in survival in hostile environments and in visceralization; even when host body temperature increases, the protein allows the parasite [14] to remain viable and multiply [14]. The protein also shares homology with the S antigen expressed by *Plasmodium falciparum*, and anti-A2 antibodies are detected in more than 90% of patients with active visceral leishmaniasis, indicating high antigenicity and underscoring its significance as a critical target for vaccine development or therapies against leishmaniasis [11].

When the antigen was administered to a group of four mice to evaluate protection against 2 × 10^8^ *Leishmania donovani* promastigotes, there was an 89% reduction in hepatic parasitic load compared with the control group. There was also an increase in specific antibodies and in the production of IFN-γ and IL-4, which are determining factors for resistance to the parasite [15]. For this purpose, a dose of 10 µg of recombinant A2 protein was used in the initial application, followed by 5 µg for the subsequent two boosters, with three-week intervals between them [15].

The vaccine was developed by the Laboratory of Cellular and Molecular Immunology at the Universidade Federal de Minas Gerais (UFMG) and was marketed in Brazil until 2023 by Ceva Saúde Animal Ltda. When administered to dogs, it stimulates immune mechanisms that help in fighting the parasite. To achieve this, it should be administered starting at four months of age to dogs seronegative for leishmaniasis, in three doses with twenty-one-day intervals between them, followed by an annual booster on the date of the first dose, as recommended by the manufacturer. It is important to note that after this protocol some animals showed systemic reactions, including apathy, anorexia, and hyporexia, in addition to more common localized reactions such as pain, lameness, and edema, which may last up to five days after the third dose [9]. However, these reactions are considered acceptable, given that, even with a parasite load in the bone marrow, the onset of clinical signs related to the disease can be delayed by up to one year post-infection, which did not occur with unvaccinated animals. Unvaccinated animals may present clinical signs between three and six months post-infection or more [10].

The delay in clinical signs may be associated with the vaccine’s ability to induce polarization of the immune response toward the Th1 type, which is considered effective in combating the parasite in dogs [16]. This response is characterized by decreased production of IL-10 from lymphocytes and antigen-presenting cells (APCs), together with a strong and sustained response mediated by CD4+ Th1 and CD8+ lymphocytes that produce high concentrations of IL-2, IFN-γ, and TNF-α [17]. In a study involving twenty-eight male and female dogs of various breeds from Ouro Preto, a city in the state of Minas Gerais, Brazil, divided into four groups, vaccination with Leish-Tec^®^ was associated with increased CD8+ IFN-γ+ lymphocytes and CD4+ cells [17]. Additionally, the antigen stimulated increased production of IFN-γ and IL-4 and a rise in highly specific IgG1 and IgG2 antibodies in these animals [10].

The type of immune response may also contribute to the lower parasitic load observed in dogs vaccinated with Leish-Tec^®^ compared with unvaccinated dogs. This reduction helps decrease the rate of sand fly infection, as vaccinated and clinically healthy animals generally exhibit little or no ability to transmit *Leishmania* to the vector when tested by xenodiagnosis [9].

In Brazil, after its proven effectiveness as an individual protection method for dogs, Leish-Tec^®^ was licensed for use in 2008 by the Ministry of Health of the Brazilian federal government. However, due to the antigen presenting amounts lower than the minimum limit established by the Ministry of Agriculture and Livestock, several batches of the vaccine were recalled, and its commercialization in the country was suspended from May 2023 [18]. In a statement, the company mentioned that the vaccine, registered by the Ministry of Agriculture and validated by the Ministry of Health, faced technical issues, prompting an investigation to determine the cause [19]. Ceva also mentioned that the primary cause of protein destabilization remains unknown and that the investigation process may take time, assuring that no harm to the health of vaccinated dogs is expected [20].

Even after the technical issues were addressed, there has been reluctance among veterinarians in Brazil to use the vaccine. Many severe side effects have been reported, including allergic reactions and anaphylactic shock, which can be attributed to the adjuvant used in the immunogen’s production. Saponins, whether natural or synthetic, are employed in various products for different purposes due to complex structures that result in diverse chemical, physical, and biological properties [21]. Nevertheless, when administered inappropriately or in excessive amounts, they can lead to intense side effects, including hemolytic activities [22,23]. A disproportion between the adjuvant amount and the Leish-Tec^®^ vaccine antigen and its excipients might explain the increased side effects of the immunogen. If this is confirmed, it will further fuel professionals’ distrust regarding the vaccine, potentially complicating its reinstatement as a method of personal protection against CVL in the country. Moreover, during its commercialization period, there was no recommendation from Brazilian ministries for its use as a public health measure, as it did not significantly impact the incidence of canine visceral leishmaniasis when administered to a larger number of dogs, nor did it affect human cases of the disease [24]. Additionally, its use was confined to registered veterinary clinics and was available at a relatively high cost, which also hindered widespread use.

When tested on a diverse population of dogs from an endemic area with high transmission rates in Brazil, the Leish-Tec^®^ vaccine demonstrated an efficacy of 71.4% based solely on parasitological tests and 58.1% when considering parasitological tests associated with xenodiagnosis. However, it did not show a significant reduction in the transmission of the parasite from dogs to sand flies when analyzed by PCR after xenodiagnosis [25]. Similar findings were reported when dogs tested exhibited high production of anti-A2 antibodies; despite this, 43% of the dogs developed the disease over the evaluation period, indicating only a modest effectiveness of 35.7% [24].

These antibodies, specific against the A2 antigen, are present to varying degrees in dogs immunized with Leish-Tec^®^, decreasing six months after immunization but increasing considerably after the annual booster, highlighting the importance of booster doses [26]. Importantly, these antibodies do not cross-react with antibodies against the *Leishmania* promastigote antigen (LPA) and rK39, antigens used in the main diagnostic methods recommended by the Ministry of Health [24]. The study by Campos, et al. [27] showed that dogs vaccinated with Leish-Tec^®^ do not test positive in the double migration or double path immunochromatographic device (DPP) or in the enzyme immunoassay (EIE), unlike unvaccinated and naturally infected dogs, which tested positive in both tests. Thus, it is possible to differentiate, through antibodies, vaccinated animals from infected ones, as the latter will produce antibodies against different antigens of the parasite. This distinction is crucial to avoid unnecessary treatments and euthanasia [28].

In terms of public health measures, the identification of dogs infected by *L. infantum* is crucial, as it marks the first step toward implementing disease control measures in these animals. In Brazil, this identification is carried out through various serological tests in line with recommendations from the Ministry of Health.

Regarding the duration of immunity conferred by the vaccine, some research suggests that it can last for one to two years after vaccination, while other studies indicate that immunity can be maintained for up to four years. For this reason, it is vital that the booster dose be administered at the correct time to increase the chances of effectively controlling the parasite. However, immunity can vary depending on factors such as age, immunological status, previous exposure to the parasite, and several other conditions inherent to the host. Therefore, it is not possible to guarantee that the vaccine is 100% effective against *Leishmania* spp., as is the case with all immunogens produced against any disease.

The use of the vaccine as immunotherapy is also a topic of discussion. Although not approved for use as immunotherapy, some studies suggest potential benefits. In an analysis of data involving 250 animals vaccinated with Leish-Tec^®^ and 245 animals in the control group, all infected by *L. infantum* and asymptomatic, three doses of the vaccine or a placebo were administered at fourteen-day intervals. It was concluded, after nine months of follow-up, that the risk for clinical progression of the disease and mortality was lower in vaccinated animals less than six years of age compared with the control group mentioned above [29]. The same research group also analyzed safety in asymptomatic dogs and found localized adverse effects in animals that received the vaccine, with an occurrence rate of 3.09%, but without any more severe reactions [30]. Both studies therefore indicated that its use as an immunotherapy is safe and effective and can be applied to clinically healthy infected animals to reduce the chance of progression of clinical signs and the possibility of reducing transmission of the parasite from dogs to the vector [29,30]. However, there are still no vaccines registered for use as immunotherapy, and before they can be indicated for this purpose, new studies need to be conducted to verify the response in animals followed for a longer period. Furthermore, long-term studies are also needed in animals that present the clinical picture of the disease to prove that its use can be approved as an effective treatment against CVL.

## 3. CaniLeish^®^

The CaniLeish^®^ vaccine (Virbac, Carros, France) is composed of 100 µg of purified secreted–excreted *L. infantum* antigens (LiESP), produced using a technology patented by the Recherche pour le Développement (IRD) [31], combined with 60 µg of the saponin adjuvant *Quillaja saponaria* (QA21) [32].

Initially, these antigens were tested along with the adjuvant muramyl dipeptide (MDP) in eighteen beagle dogs, ten males and eight females, aged between one and six years [33]. The animals were divided into four groups: one group of three animals was inoculated with 200 µg of LiESAp with MDP, another group of three animals received 50 µg of LiESAp with MDP, and a third group consisting of six animals was inoculated with 100 µg of antigen plus adjuvant. These groups were compared to a control group of six animals, inoculated with 200 µg of MDP and infected with 10^8^ metacyclic promastigotes of *L. infantum*, eight months after the second vaccination [33]. The study demonstrated that this vaccine protected 100% of dogs, which remained negative in parasitological tests for up to fourteen months post-infection [33].

With such positive results, another study was conducted involving four hundred and fourteen dogs naturally exposed to the parasite in an endemic area for leishmaniasis in France. These dogs were inoculated with two doses composed of 100 µg of LiESAp and 200 µg of MDP subcutaneously and monitored for a period of two years, corresponding to two periods of sand fly activity (occurring from May to October). Clinical signs were recorded, and blood samples were collected before vaccination and at six and twelve months post-vaccination [34]. The development of the disease was monitored using an indirect immunofluorescence reaction (IFAT) and IgG2-specific antibodies using an enzyme-linked immunosorbent assay (ELISA), in addition to monitoring the leishmanicidal activity of macrophages and nitric oxide production [34]. As a result, 98.2% of vaccinated dogs presented anti-LiESAp IgG2 antibodies after six months of immunization, and the leishmanicidal activity of macrophages increased significantly after vaccination, along with the production of nitric oxide, leading to a decrease in the infection rate from 6.86% to 0.61% [34]. These results can be attributed to the immune response conferred by the immunogen on dogs.

In a study by Bourdoiseau, et al. [35] involving eighteen dogs, nine males and nine females, divided into four groups, one group consisted of six animals vaccinated with MDP, while the other three groups, comprising three animals each, were vaccinated with LiESAp plus MDP. These animals received two subcutaneous injections with a three-week interval between them. Two of the groups were infected with 10^8^ *L. infantum* metacyclic promastigotes two months after vaccination, while the other two groups were challenged eight months after vaccination. Both groups were monitored by isolating parasites from bone marrow aspirates and presented negative results for eight months after challenge. The ELISA technique demonstrated a significant increase in anti-LiESAp IgG2 at two and eight months after vaccination.

When tested by Moreno, Vouldoukis, Martin, McGahie, Cuisinier and Gueguen [32], the CaniLeish^®^ vaccine, together with its adjuvant component at that time, stimulated a specific CD8+ cell response against *Leishmania* in twenty beagle dogs. These dogs were vaccinated with three doses at intervals of twenty-one days, resulting in an increase in IFN-γ concentrations, NO_2_, IgG1 and IgG2. This demonstrated a mixture of Th1 and Th2 responses, with a predominance of Th1 response, which is responsible for resistance to the parasite. Additionally, macrophages from dogs vaccinated with CaniLeish^®^ had an increased capacity for parasite phagocytosis, aiding in combatting infection. These immune responses persisted for up to a year after vaccination, validating the expectation that the vaccine can protect animals for a long period of time [36].

One year after vaccination, even before the annual booster, Martin, et al. [37] challenged dogs with a suspension of 10^8^ parasites/mL intravenously to monitor the appearance of possible clinical signs and parasite load in the bone marrow. It was observed that the immunological profile of vaccinated dogs continued to be polarized toward Th1, significantly reducing the risk of disease progression, as well as the parasite load in the bone marrow [37].

A study was conducted in two areas of high transmission of the parasite, with twenty-three dogs vaccinated according to the protocol recommended by the manufacturer and twenty-two dogs in the control group in Barcelona, Spain, and the same number in the province of Naples, Italy, totaling ninety animals seronegative for *Leishmania* [38]. The dogs were evaluated every three months, and the main adverse effects of the vaccine were edema and local pain. This study demonstrated that 72% of dogs in the control group had the infection, and 90% had serological evidence of exposure, with one-third showing active infection up to two years post-exposure, confirming that the dogs in the experiment were exposed [38]. Furthermore, there was no significant difference between the control group and the vaccinated group in terms of positive polymerase chain reaction (PCR) results, indicating that the vaccine is not capable of preventing infection or migration of the parasite into tissues. However, with respect to clinical signs of the disease, vaccine efficacy was 68.4% [38].

A study by Montoya, et al. [39] also demonstrated the safety of the vaccine, in which, out of three hundred and fourteen dogs observed, only twenty showed adverse reactions. Eight of these showed clinical signs within thirty minutes post-vaccination, such as anaphylactic reactions, facial erythema, and local edema at the injection site, while others presented adverse reactions hours or days after vaccination, such as apathy, vomiting and/or diarrhea, and fecal or urinary incontinence.

To evaluate the ability of vaccinated animals to transmit the parasite to the vector, a study involving ten dogs at different stages of infection by *L. infantum* found that despite being infected, the reduction in the parasite load leads to a reduction in the infectivity of the dogs. Therefore, if associated with other prophylaxis methods, such as topical insecticides, vaccination can help reduce cases in endemic areas [40].

Based on initial results, CaniLeish^®^ was licensed in European Union countries from 2011 onwards for *Leishmania*-seronegative dogs from six months of age, administered in three doses with twenty-one-day intervals between them, with annual booster, according to the manufacturer’s recommendation (Virbac, France). Nevertheless, while providing some level of protection, the vaccine does not demonstrate significant efficacy in preventing infection to the extent of reducing the number of canine cases within up to one year after vaccination in an endemic area. Therefore, it is not recommended for use as the sole collective protection measure in endemic areas and should always be supplemented with the use of repellents and insecticides. For instance, collars impregnated with 4% deltamethrin (Scalibor^®^) and collars containing imidacloprid and flumethrin (Seresto^®^) have shown efficacy rates of 61.8% and 88.3%, respectively. Thus, like these collars, the CaniLeish^®^ vaccine should be used in conjunction with repellents and insecticides to enhance overall protection [41].

Despite providing some protection, the CaniLeish^®^ vaccine showed limited efficacy in preventing infection over time, particularly in endemic areas. Consequently, after more than a decade of commercialization, the marketing authorization for CaniLeish^®^ was officially withdrawn at the request of the manufacturer in 2023, marking the end of its availability in the European market [42].

For issues related to interference with serological tests, Oliva, Nieto, Foglia Manzillo, Cappiello, Fiorentino, Di Muccio, Scalone, Moreno, Chicharro, Carrillo, Butaud, Guegand, Martin, Cuisinier, McGahie, Gueguen, Cañavate and Gradoni [38] found that using IFAT to test for *L. infantum* infection in dogs vaccinated with CaniLeish^®^ is not recommended. These animals showed positive titers due to antibodies induced by the vaccine, which are not specific to the vaccine antigen and cannot be differentiated from infected dogs [38]. This was also demonstrated in the study by Montoya et al. [39], which detected low antibody titers through IFAT even after twelve months of vaccination, recommending that, for the diagnosis of CVL in vaccinated dogs, it would be more prudent to use molecular methods. However, this same study indicated that the Speedleish^KTM^ test was not able to detect vaccine antibodies in most dogs tested, except for two animals, which had detectable antibody titers and were negative for *Leishmania* spp. in molecular tests [39]. The researchers therefore concluded that it is necessary to carefully interpret test results before considering animals that have been vaccinated as infected, as this could be a false positive due to the vaccine [39].

Another study by Velez, Domenech, Cairó and Gállego [28] showed that dogs vaccinated with CaniLeish^®^ present high titers of antibodies. These antibodies are detectable by the enzyme-linked immunosorbent assay using *L. infantum* crude antigen (ELISA-CTLA), especially one month after vaccination, in which 74.1% of the animals were positive. This also makes it difficult to differentiate positive dogs from those vaccinated using this diagnostic method. Therefore, there are still no serological diagnostic methods available that can fully differentiate animals vaccinated with CaniLeish^®^ from infected animals, because the antibodies induced by the vaccine, even at low to medium titers, are the same as those induced by natural infection, which makes the interpretation of laboratory tests difficult [43]. This is a reasonable difficulty to be considered when discussing the use of this vaccine in Brazil.

The impossibility of differentiating infected from vaccinated animals through serological tests is an obstacle, as disease control in endemic areas relies on these tests to implement preventive and control actions [44].

Although the CaniLeish^®^ vaccine initially offered a tool to combat canine leishmaniasis, its limited efficacy and the challenges in distinguishing vaccinated from infected animals through serological testing may have contributed to its withdrawal from the European market. These are important factors to consider regarding the use of CaniLeish^®^ and other vaccines in regions such as Brazil, where accurate diagnosis is essential for effective disease control.

## 4. Leti-Fend^®^

The LetiFend^®^ vaccine features a chimeric protein, known as the Q protein. It comprises five antigenic fragments derived from four different *L. infantum* proteins, including LiP2a, LiP2b, LiP0, and H2A, without the addition of an adjuvant [45]. These antigens were initially evaluated as potential targets for serodiagnosis in dogs infected with *L. infantum*, using canine sera samples from various groups, including infected dogs, negative controls, and dogs infected with other pathogens [46]. This study revealed promising results, indicating that the Q protein can serve as an antigen for serological diagnostic tests, exhibiting high sensitivity and specificity.

Subsequently, the Q protein was investigated as a vaccine candidate. In an initial study involving mice, a vaccine formulation containing 2 µg of the Q protein and 50,000 PFU of live Bacillus Calmette–Guérin (BCG) was administered to BALB/c mice, which were challenged by *L. infantum* two weeks after vaccination. As a result, there was a significant reduction (80%) in parasite load in the spleen post-infection [47].

Encouraged by these findings, a similar vaccination protocol was carried out in ten beagle dogs, which demonstrated a vaccine efficacy of 90%, with nine vaccinated dogs showing elimination of parasites upon infection [47]. However, BCG adjuvant was associated with adverse effects in dogs, rendering it unsuitable for commercial vaccine use. Consequently, the Q protein was tested with alternative adjuvants, such as gMDP, Al(OH)_3_, ISCOMatrix C, and *Propionibacterium acnes*, in subsequent studies [48]. Despite inducing antibody production, all groups tested positive for *Leishmania* spp. in bone marrow and lymph node cultures, suggesting that BCG may have a greater impact against the parasite compared to the antigen itself [48].

A separate study demonstrated that the Q protein, when administered without adjuvant, could still induce the production of IgG2-type antibodies and effectively reduce parasitic load in vaccinated dogs’ organs [49]. To this end, this study was carried out with twenty-one beagles, including twelve females and nine males aged one to two years, vaccinated with 100 µg of lyophilized protein: one group of seven animals received only one dose, and another group of seven animals received two doses with a twenty-one-day interval between them, in addition to the control group that received placebo [49]. Sixty days after vaccination, the dogs were infected with 5 × 10^5^ *L. infantum* promastigotes. Clinical signs were evaluated for sixty days post-infection, in addition to serological evaluation by IFAT and ELISA, parasite load by PCR, delayed-type hypersensitivity (DTH), and Western blotting [49].

The immune response elicited by LetiFend^®^ predominantly involves IgG2 antibodies, which exhibit increased concentrations between days fourteen and twenty-eight post-vaccination, as demonstrated in studies [45]. Additionally, LetiFend^®^ has been shown to induce the production of IFN-γ and IL-4, particularly when the Q protein is combined with CpG-ODN, leading to a reduction in parasite load in the liver and spleen of mice and providing long-term protection [50]. Furthermore, LetiFend^®^ has been found to stimulate an increase in proteins from the complement system and the serpin family, which play crucial roles in immune regulation processes and host protection against parasitic diseases [51].

In a study involving forty-four dogs challenged with *L. infantum*, LetiFend^®^ demonstrated its ability to stimulate protective mechanisms against visceral leishmaniasis [51]. Subsequently, LetiFend^®^ was licensed in the European Union in February 2016 and is administered in a single dose with annual boosters in dogs over six months of age [44]. This single-dose protocol enhances owner adherence to vaccination because it eliminates the need for multiple doses at specific intervals. Additionally, it simplifies public health efforts, especially in vaccinating stray dogs, as they do not require ongoing monitoring to ensure compliance with the vaccination schedule. This streamlined approach reduces management costs and the logistical challenges associated with multi-dose protocols, despite the requirement for an annual booster.

The phase III study of the vaccine used five hundred and forty-nine animals of different breeds aged between six months and fourteen years for seven hundred and thirty days. These animals were divided into groups vaccinated with LetiFend^®^ and groups vaccinated with a formulation without the Q protein, naturally exposed to the parasite, and tested using PCR, ELISA, and cytology of lymph nodes. Bone marrow samples were analyzed to determine whether they tested positive for *L. infantum* [45]. This research showed a significant difference between the appearance of clinical signs in vaccinated dogs and those in the control group, with an efficacy of 72% in preventing clinical signs of CVL and with a reduction in the probability of confirmed cases of the disease.

Iniesta, et al. [52] conducted a study that tested ten six-month-old beagle dogs, consisting of four males and six females, vaccinated with LetiFend^®^. These dogs were compared to twenty dogs from two control groups of the same age and breed. The control groups consisted of healthy dogs and infected dogs, respectively. All animals underwent testing to detect antibodies against *L. infantum* using the in-house soluble *Leishmania* antigen (SLA) ELISA, in-house *Leishmania* ELISA, Leiscan^®^ (Esteve Veterinaria, Laboratorios Dr. Esteve SA, Barcelona, Spain), Ingezim^®^ Leishmania (INGENASA, Madrid, Spain), and the rapid tests Kalazar Detect™ (InBios International, Seattle, WA, USA), Snap^©^ *Leishmania* (IDEXX, Inc., Westbrook, ME, USA), Speedleish KTM (BVT Virbac Group, La Seyne sur Mer, France), WITNESS^®^ *Leishmania* (Synbiotics Corporation, Kansas City, MO, USA), and Uranotest *Leishmania* (Urano^®^Vet, Barcelona, Spain). In the study cited, vaccinated dogs that remained uninfected according to lymph node and bone marrow smears were also negative in routine serological assays during the first 28 days after vaccination. However, this finding should not be interpreted as evidence that serology is a reliable marker of protection. Seropositivity in canine leishmaniasis is generally associated with disease progression rather than with immunity, and antibody responses elicited by vaccination may vary depending on the formulation. Indeed, some vaccines (e.g., CaniLeish^®^) are known to induce antibody responses that can compromise the interpretation of standard serological tests used in endemic areas. [52].

Therefore, it is possible to differentiate vaccinated and healthy dogs from infected dogs, which is highly relevant for the implementation of vaccination as a public health measure. Since this differentiation avoids unnecessary treatments and euthanasia, LetiFend^®^ shows an important advantage. If its efficacy is further confirmed under endemic conditions in Brazil, the vaccine could be considered as part of prophylactic measures against CVL in the country.

A study by Fernández Cotrina, Iniesta, Monroy, Baz, Hugnet, Marañon, Fabra, Gómez-Nieto and Alonso [45] demonstrated that there was no significant difference between the vaccinated and placebo groups regarding the presence of parasites in lymphoid organs detected by PCR. For this reason, more studies are needed to prove that the vaccine can be effective in preventing the transmission of the parasite before it is implemented in places with a high transmission rate.

## 5. NeoLeish^®^

The NeoLeish^®^ vaccine is composed of CpG DNA islands encoding the *L. infantum* activated protein kinase C receptor analogue (LACK) gene [53]. This antigen, analogous to the mammalian receptor for activated protein kinase C (RACK), comprises WD40 repeat motifs, associated with the parasite’s regulatory functions, and is localized in the kinetoplast [54].

It was initially tested for the protection of BALB/c mice against *L. major* [55]. In this study, twelve female mice per group aged six to eight weeks were immunized subcutaneously with either 100 µg of LACK DNA or 50 µg of LACK protein, with or without IL-12, compared to a control group immunized only with plasmid DNA, and subsequently challenged with 10^5^ promastigotes of *L. major* [55]. The results showed that LACK DNA conferred protection to the mice, lasting up to twenty weeks post-infection [55].

It was later tested by Pinto, et al. [56] in female BALB/c mice aged four to six weeks. Three groups of five animals were immunized twice intranasally with 10 µg of *L. amazonensis* antigens, 10 µg of LACK, or 30 µg of LACK DNA, compared to a control group of five animals immunized with 30 µg of plasmid DNA. The animals were challenged up to seventeen weeks after the second dose with 10^5^ promastigotes of *L. amazonensis* [56]. It was observed that intranasal immunization with LACK was not effective against *L. amazonensis* infection when animals were evaluated in the short term [56].

Gomes, et al. [57] evaluated the antigen administered in BALB/c mice via the same route and concluded that protective immunity was triggered by the vaccination. The study involved a group of six animals aged six to eight weeks vaccinated with 15 µg of pCI-neo-LACK in two intranasal doses with a seven-day interval [57]. These animals were compared to two control groups, each consisting of six animals that received PBS and the pCI-neo plasmid. All groups were challenged with 10^7^ promastigotes of *L. chagasi* one week after the second dose [57]. After thirty days of infection, it was observed that the vaccinated animals did not exhibit lethargy, unlike the non-vaccinated animals. Furthermore, vaccinated animals produced higher levels of IFN-γ, lower levels of IL-10, and significantly higher anti-*Leishmania* antibody levels as identified by ELISA [57]. The authors concluded that intranasal vaccination with pCI-neo-LACK stimulates protective immunity against visceral leishmaniasis in mice [57].

The pCI-neo-LACK plasmid (DNA-LACK) was also tested in twenty dogs aged between eighteen months and 4.5 years, divided into four groups [58]. Three of these groups were infected with 10^8^ promastigotes of *L. infantum* after immunizations, receiving either two subcutaneous doses of 100 µg of plasmid with a fifteen-day interval, a single plasmid dose followed by a second dose of pfu of recombinant vaccinia virus (rVV-LACK), or no dose [58]. A control group, which did not receive the vaccine or infection, was also included [58]. At the end of the experiment, after seventeen months, four animals from the DNA-LACK group exhibited clinical signs, while only one dog from the DNA-LACK + rVV-LACK group showed signs of VL. Regarding the presence of anti-*L. infantum* antibodies measured by DAT, all dogs in the DNA-LACK group were positive, whereas in the DNA-LACK + rVV-LACK group, only two were positive. Parasite loads in the liver and spleen were lower in the DNA-LACK group compared to the positive control group, although all animals still had a detectable load. However, in the DNA-LACK + rVV-LACK group, only two animals had detectable liver parasite loads and one had a spleen parasitic burden, suggesting more promising results in this group. It was concluded that the LACK antigen, depending on its delivery method, shows promise in protecting against canine VL.

Building on these findings, Alonso, Alcolea, Larraga, Peris, Esteban, Cortés, Ruiz-García, Castillo and Larraga [53] conducted a preclinical trial assessing the response of fifteen beagle dogs to 200 µg of pPAL-LACK administered intranasally in two doses, thirty days apart. This group was compared to a control group of fifteen dogs. Thirty days after the first dose, the animals were challenged with 10^8^ promastigotes of *L. infantum* and monitored for up to three hundred days post-infection [53]. The study concluded that clinical signs were reduced in 60% of vaccinated dogs, with the remaining vaccinated animals exhibiting milder symptoms, while the control group showed worsening symptoms [53]. Regarding parasite load in the spleen and liver, evaluated by qPCR, the vaccinated group showed a reduction compared to the control, and the number of amastigotes in bone marrow decreased by 92% [53]. The control dogs exhibited high IgG1 and IgG2 titers, whereas vaccinated dogs showed high IgG2 and low IgG1 titers, indicating a polarization toward a Th1 immune response in vaccinated animals, confirmed by higher CD4+ T-cell proliferation in lymph nodes and spleen, with higher levels of IFN-γ and lower IL-10 compared to non-vaccinated animals [53]. Therefore, these results indicate that LACK antigen administered intranasally provides high protection. These studies led to the recommendation for the use of the vaccine as an individual protection measure for dogs by the European Commission in November 2022 (EMA/CVMP/858971/2022).

NeoLeish^®^ is available in a liquid formula, containing 212.5–250 µg of pPAL-LACK plasmid in 1 mL of solution, which should be stored at temperatures between 2 °C and 8 °C. The recommended protocol involves an initial intranasal dose starting at six months of age, followed by a second dose two weeks after the first [59]. A booster is required, administered six months after the first vaccination [59].

According to the European Medicines [59], NeoLeish^®^ reduces the risk of developing infection and clinical signs of VL after exposure to *L. infantum* from six months of age. Additionally, the NeoLeish^®^ vaccine does not interfere with serological diagnostic tests, such as those using the rK39 antigen, recommended by the Ministry of Health [24]. This is because the LACK protein encoded by the vaccine’s genetic material and expressed by the dogs’ cells is highly specific and different from the proteins detected in these tests. Thus, it is possible to safely differentiate vaccinated dogs from infected ones, without the risk of false positives related to vaccination [59]. This is a significant advantage for vaccine implementation in endemic countries that advocate euthanasia of infected animals as a control measure for VL, as the risk of mistakenly euthanizing vaccinated animals due to diagnostic errors will be minimal.

## 6. Discussion

Currently, there is a global imperative to develop an effective vaccine against canine visceral leishmaniasis. This imperative stems from the recognition that successfully preventing the disease in dogs could result in a significant decrease in human cases, given the zoonotic transmission of *L. infantum* [60]. To accomplish this objective, vaccines must not only prevent the onset of clinical signs but also reduce the parasitic load in animals, thereby diminishing the transmission of the parasite to sand flies.

As of May 2023, four vaccines were commercially available globally. In Europe, CaniLeish^®^, LetiFend^®^, and NeoLeish^®^ were still available, while in Brazil, Leish-Tec^®^ was commercialized but discontinued due to production issues. In October 2023, CaniLeish^®^ was officially discontinued by the European Commission. It is important to emphasize that, although the Leish-Tec^®^ and CaniLeish^®^ vaccines have contributed valuable data on immunogenicity and safety, they are no longer commercially available. Their discontinuation reflects regulatory or commercial limitations, and there is currently no prospect of their reintroduction in the near future. Therefore, their relevance today lies primarily in the lessons learned for the development of next-generation vaccines, rather than in their direct application in veterinary practice. The main characteristics of the vaccines are summarized in Table 1. The recommended age for starting the vaccination protocol with CaniLeish^®^, LetiFend^®^, and NeoLeish^®^ is from six months, while Leish-Tec^®^ can be administered from four months, which can be considered advantageous. Initiating the vaccination protocol earlier, particularly in endemic countries with year-round transmission like Brazil, could be crucial for protecting dogs, as they may have contact with the parasite from birth.

CaniLeish^®^ and Leish-Tec^®^ require three doses to complete the protocol, while LetiFend^®^ is administered in a single dose, providing a cost–benefit advantage. This reduces the overall cost of the protocol, and owners do not need to visit the veterinarian multiple times for vaccination, thereby potentially increasing vaccination adherence.

NeoLeish^®^, on the other hand, requires two doses administered intranasally, which could present a less painful and less stressful alternative for animals compared with subcutaneous injections. This method may also improve acceptance by veterinarians and pet owners, contributing to better adherence to the vaccination protocol and overall coverage. Nevertheless, NeoLeish^®^ requires a booster every six months, which is a significantly shorter interval compared with the booster schedules of other vaccines. This requirement may pose a challenge, as pet owners might be reluctant to return to the veterinarian in such a brief timeframe.

While LetiFend^®^ and NeoLeish^®^ offer advantages in terms of administration and immunization, they are not yet recommended as public health measures, as there is no clear evidence of a significant reduction in canine or human cases on a large scale. Similar concerns exist for CaniLeish^®^ and Leish-Tec^®^, as studies have yet to demonstrate robust efficacy in preventing parasite transmission or reducing human and canine cases through inherited immunity. Although some vaccines reduce parasite burden in individual animals, there is currently no solid evidence that they confer a population-level impact or herd effect. The lack of demonstrated reductions in canine or human cases at a community scale limits the use of these vaccines as public health interventions. Therefore, in endemic regions, vaccination should be considered a complementary measure rather than a stand-alone strategy, with established vector control and environmental management remaining essential, as does the use of deltamethrin collars, which have been proven effective in preventing infection [61]. Additionally, all four vaccines require annual boosters to maintain adequate levels of protection.

Studies have shown vaccine efficacy rates of 71.4% for Leish-Tec^®^, 68.4% for CaniLeish^®^ [38], and 72% for LetiFend^®^ [45] in preventing the onset of clinical signs of the disease. Importantly, NeoLeish^®^ demonstrated a 60% reduction in clinical signs and a 92% reduction in the parasitic load in the bone marrow [53]. The details of the studies are described in Table 2. However, regarding the prevention of *L. infantum* infection, studies have not yet shown satisfactory protection levels for either immunogen, including NeoLeish^®^, which, despite its efficacy in reducing clinical signs and parasitic load, does not guarantee complete prevention of infection. Despite some vaccines reducing parasitic load in individual animals, there is currently no strong evidence that they confer a population-level impact or herd effect. The absence of demonstrated reductions in canine or human cases at a community scale limits the use of these vaccines as public health interventions. Therefore, in endemic regions, vaccination should be considered as a complementary measure rather than a standalone strategy, with established vector control and environmental management remaining essential.

However, regarding the prevention of *L. infantum* infection, studies have not yet demonstrated satisfactory levels of protection for any of the immunogens, including NeoLeish^®^. Despite its effectiveness in reducing clinical signs and parasite load, NeoLeish^®^ does not guarantee complete prevention of infection. Although some of these vaccines reduce parasite load in individual animals, there is no solid evidence that they confer a population-level impact or herd effect. The lack of demonstrated reductions in canine or human cases at a community level limits the use of these vaccines as public health interventions. Therefore, in endemic regions, vaccination should be considered a complementary measure rather than a stand-alone strategy, with established vector control and environmental management remaining essential.

It is worth noting that there is no consensus among researchers regarding the characteristics of an appropriate challenge for vaccine testing. Variables such as the evolutionary stage of the parasite used, the dose administered, or the route of administration are not clearly defined. These variables are crucial in challenge trials, as they can influence the patient’s response to infection, potentially suppressing any protective effects of immunogens. Although current vaccines have demonstrated efficacy in reducing clinical signs and parasite load, variability in results across studies highlights methodological limitations. Differences in challenge models, parasite doses, routes of administration, and population sizes make direct comparisons challenging [62]. Furthermore, excluding animals from field trials due to low infection rates can artificially inflate efficacy estimates. These factors reinforce the need for standardized protocols in vaccine evaluation, which would allow for more robust comparisons and guide the development of next-generation vaccines.

It is important to note that the efficacy results of LetiFend^®^ should be interpreted with caution due to methodological aspects of the main trial. In the pre-clinical challenge, animals were exposed to 10^5^ promastigotes per dog, whereas the NeoLeish^®^ trial used 10^8^ promastigotes, a difference that may have directly influenced efficacy outcomes. Furthermore, in the pivotal field study of LetiFend^®^, 19 kennels were initially included, but 17 were excluded because of the low incidence of natural infection. As a result, the final efficacy value of 72% was calculated from only 50 dogs, which considerably reduces the robustness of the conclusions. These factors should be acknowledged when comparing results across vaccines.

A Bongiorno, Paparcone, Foglia Manzillo, Oliva, Cuisinier and Gradoni [40] suggest that CaniLeish^®^ can reduce the parasite load in dogs, thereby decreasing their capacity to transmit the parasite to sandflies. However, the vaccine interferes with serological tests, as vaccination-induced antibodies cannot be distinguished from those produced during an active *Leishmania* infection. This has important implications in endemic areas, where serology is used to guide decisions regarding treatment or euthanasia. In Brazil, for example, the Ministry of Agriculture and Livestock recommend immunochromatographic and ELISA tests for screening and confirming *Leishmania* infection. If vaccinated and infected animals cannot be distinguished, the accuracy of these tests would be compromised, necessitating the use of more specific methods, such as molecular tests. These methods, however, may be impractical in some regions due to high costs and the need for specialized laboratories, which are often located far away. For these reasons, the introduction of CaniLeish^®^ in endemic countries must be carefully evaluated. Considering diagnostic cross-reactivity is crucial, as misclassifying vaccinated animals as infected can lead to unnecessary interventions and undermine public confidence in control programs. Leish-Tec^®^, LetiFend^®^, and NeoLeish^®^, by contrast, do not show cross-reactivity in serological tests, allowing vaccinated and infected animals to be distinguished—a key factor in avoiding unnecessary treatment or euthanasia.

The advantages and disadvantages of each vaccine, summarized in Table 3, should be carefully considered when planning their commercialization or reintroduction in countries endemic for canine visceral leishmaniasis, such as Brazil. Furthermore, these characteristics can serve as a basis for the development of new vaccine antigens with higher protection rates, better cost-effectiveness, and fewer adverse effects. Such adverse effects are often related to the presence of adjuvants in vaccine formulations; therefore, vaccines containing these substances must be carefully weighed against their potential benefits. Studies have shown that certain adjuvants, such as BCG, can elicit strong immune responses but also cause significant adverse effects, making them unsuitable for commercial use [47]. Furthermore, immunogens that use saponin as an adjuvant can, when administered in inadequate amounts, trigger hemolytic events or other serious side effects, which can directly affect owner compliance and, consequently, the feasibility of mass vaccination campaigns. Therefore, comprehensive post-marketing surveillance and thorough assessment of risks and benefits are essential to ensure vaccine safety, especially in large or high-density dog populations.

Despite the valuable lessons learned from the development and use of existing vaccines, significant gaps remain in our understanding of protective correlates against *Leishmania* infection. Identifying reliable immunological markers that predict long-term protection is essential for guiding the design of next-generation vaccines and for evaluating their efficacy in both preclinical and field settings. Emerging vaccination strategies, such as DNA vaccines, viral-vectored platforms, and intranasal formulations, offer promising avenues to enhance immunogenicity, improve safety profiles, and potentially reduce the number of doses required. Additionally, multivalent vaccines targeting multiple antigens simultaneously may provide broader protection against diverse parasite strains, while also mitigating the limitations associated with single-antigen immunogens. Beyond the vaccine formulation itself, integrating immunization with established vector control measures—such as insecticide-treated collars, environmental management, and community engagement—could substantially increase the overall effectiveness of control programs. Such integrated approaches may not only reduce clinical disease in dogs but also diminish parasite transmission to sandflies, thereby amplifying public health benefits. Ultimately, advancing canine leishmaniasis vaccination will require a multifaceted strategy that combines a deeper understanding of host immune responses, innovative vaccine technologies, and coordinated public health interventions to achieve meaningful and sustainable reductions in disease incidence. Therefore, the primary objective of this review is to delineate the properties of CVL vaccines, emphasizing the need for additional studies to better define their role as an individual prevention method. Additionally, it aims to improve veterinary and owner adherence to vaccination protocols and facilitate the integration of vaccines into public health measures.

## 7. Conclusions

The prevention of canine visceral leishmaniasis remains a topic requiring extensive discussion within the scientific community. Developing vaccines against parasitic agents poses significant challenges, and solutions such as Leish-Tec^®^, CaniLeish^®^, LetiFend^®^, and NeoLeish^®^ are regarded as promising avenues. However, the suspension of Leish-Tec^®^’s commercialization in Brazil and CaniLeish^®^’s in Europe has generated uncertainty among veterinarians and pet owners regarding the availability of vaccines for preventing canine visceral leishmaniasis. Consequently, acceptance of these vaccines in clinical practice may be more likely if their commercialization is reinstated. Regarding CaniLeish^®^, its interference with serological tests represents a primary drawback, potentially impeding its adoption in endemic regions. NeoLeish^®^ appears as a promising alternative, mainly because of its innovative intranasal administration and its high specificity, which avoids interference with serological diagnostic tests commonly used in endemic regions. Immunological studies have shown activation of Th1 cell subsets, supporting the importance of cell-mediated immunity in protection against *Leishmania infantum*. These results indicate that NeoLeish^®^ may trigger immune mechanisms consistent with protection, although its real impact at the population level is still uncertain. Moreover, the vaccine has been associated with reduced clinical signs and lower parasite loads, features that support its potential for broader use if future studies confirm its efficacy. A drawback, however, is the need for a booster dose every six months, which represents a limitation for large-scale application. In contrast, LetiFend^®^ does not hinder the primary diagnostic tests utilized worldwide and provides the additional advantage of being administered in a single dose. Therefore, provided that further studies confirm its effectiveness, especially in endemic regions with year-round transmission, LetiFend^®^ holds potential for combating canine visceral leishmaniasis.

## Figures and Tables

**Table 1:** Canine Visceral Leishmaniasis (CVL) vaccines currently or previously marketed worldwide.

Trade Name	Antigen/Adjuvant	Minimum Administration Age	Recommended Vaccination Protocol	Protection Percentage	Current State of Commercialization
Leish-tec^®^	Recombinant A2 protein/Saponin	4 months	3 doses at intervals of 21 days. Annual reinforcement.	71.4%	Discontinued in May 2023 in Brazil
CaniLeish^®^	LiESP/QA21	6 months	3 doses at intervals of 21 days. Annual reinforcement.	68.4%	Discontinued in October 2023 in Europe
LetiFend^®^	Q protein (Without adjuvant)	6 months	Single dose. Annual reinforcement	72%	Still on the market
Neoleish^®^	pPAL-LACK supercoiled plasmid DNA coding for LACK protein from *L. infantum*	6 months	Two doses at interval of 15 days. 6-month booster	60%	Still on the market

**Table 2:** Details on vaccine efficacy studies.

Trade Name	Study	Challenge	Sample Size	Outcome Assessment Methods	Time to Assess Outcome	Protection Rate Against Clinical Signs	Protection Rate Against Infection
Leish-tec^®^	[25]	Natural in endemic area	387 dogs used in the final efficacy analysis	Clinical signs, serology (ELISA, IFAT, KD), direct parasitology, bone marrow culture, xenodiagnosis + PCR	18 months	71.4%	58.1%
CaniLeish^®^	[38]	Natural in endemic area	90 cães	Clinical signs, serology (IFAT, ELISA), parasitology (lymph node, spleen, liver puncture), PCR and xenodiagnosis. 24 months.	24 months	68.4%	Not specified
LetiFend^®^	[45]	Natural in endemic area		Clinical signs, serology (ELISA PQ, ELISA SLA, IFAT), parasitology (lymph node and bone marrow—smear + PCR)	24 months	72%	Not specified
Neoleish^®^	[53]	Natural in endemic area	30 cães	100/5.000 Serology (ELISA), quantitative PCR, analysis of T lymphocyte subpopulations (Th1), clinical signs	12 months	60%	92%

**Table 3:** Comparative Analysis of Key Advantages and Disadvantages of CVL Vaccines.

Trade Name	Main Advantages	Main Disadvantages
Leish-tec^®^	Stimulates an immune response characterized by increased production of IFN-γ and IL-4, along with specific antibodies of the IgG1 and IgG2 types. May contribute to reducing the parasitic load in vaccinated dogs and potentially decrease the rate of sand fly infection. Does not show cross-reaction in serological tests.	Recall and suspension of commercialization due to A2 protein amounts lower than the minimum limit established by regulatory authorities. Reports of severe side effects, including allergic reactions and anaphylactic shock, potentially associated with the adjuvant used. Limited efficacy in preventing transmission of the parasite from dogs to sand flies and lack of significant impact on the incidence of CVL when administered to a larger number of dogs. Restricted availability in registered veterinary clinics and relatively high cost, limiting widespread use.
CaniLeish^®^	Induces specific antibodies against *L. infantum* antigens, potentially reducing the risk of disease progression. Demonstrated safety and effectiveness as an immunotherapy in asymptomatic dogs, reducing the chance of clinical progression and transmission of the parasite. Favorable immunological conditions that persist more stably for longer.	Limited efficacy in preventing infection, requiring additional prophylactic measures such as repellents and insecticides. Interference with serological tests, leading to challenges in differentiating vaccinated animals from infected ones, particularly in traditional serological diagnostic methods. Adverse reactions reported, including local edema and pain.
LetiFend^®^	Single-dose protocol enhances owner compliance and facilitates vaccination of stray dogs. Differentiates vaccinated and healthy dogs from infected ones in serological tests. Demonstrated effectiveness in reducing clinical signs of CVL.	Limited evidence on preventing parasite transmission. Lack of significant reduction in parasite presence in lymphoid organs detected by PCR. Potential need for additional prophylactic measures in endemic areas.
Neoleish^®^	Demonstrated effectiveness in reducing clinical signs of CVL and parasite load in bone marrow, liver, and spleen. Also reduces the risk of developing infection and clinical signs of VL after exposure to *L. infantum* from six months of age. Administered intranasally, reducing stress for animals. Does not interfere with serological diagnostic tests.	Requires booster every six months. Lack of large-scale efficacy data.

## Data Availability

Not applicable.
